# Endovaskuläre Aortenreparatur bei Endoleaks: Diagnose, Behandlung und Ergebnisse

**DOI:** 10.1007/s00117-022-01033-3

**Published:** 2022-06-23

**Authors:** Sven Rudolf Hauck, Rüdiger Schernthaner, Theresa-Marie Dachs, Maximilian Kern, Martin Funovics

**Affiliations:** 1 Abteilung für Kardiovaskuläre und Interventionelle Radiologie, Universitätsklinik für Radiologie und Nuklearmedizin, Medizinische Universität Wien, Währinger Gürtel 18-20, 1090 Wien, Österreich; 2 Zentrales Radiologie Institut – Diagnostische und Interventionelle Radiologie, Klinik Landstraße, Wien, Österreich; 3 Institut für Radiologie, Klinik Floridsdorf, Wien, Österreich

**Keywords:** Endovaskuläre Aortenreparatur, Aortenaneurysma, Endoleak, Computertomographie, Angiographie, Endovascular aortic repair, Aortic aneurysm, Endoleak, Computed tomography, Angiography

## Abstract

Sämtliche Patienten nach endovaskulärer Versorgung eines Aortenaneurysmas bedürfen einer regelmäßigen Nachkontrolle, zumeist in jährlichem Abstand. Der kontrastmittelverstärkte Ultraschall und die Computertomographie-Angiographie (CTA) sind die wichtigsten diagnostischen Modalitäten für die Erkennung von Endoleaks. Die (CTA) erlaubt eine bessere Unterscheidung der verschiedenen Endoleak-Typen. Sogenannte Hochdruck-Endoleaks (Typ I und Typ III) stellen, wenn sich nicht kurzzeitig ein Spontanverschluss zeigt, eine absolute Indikation zur Nachbehandlung dar. Typ-II-Endoleaks weisen in der Mehrzahl einen benignen Verlauf auf. Wenn kein Wachstum des Aneurysmasacks erfolgt, kann eine Nachkontrolle im gewohnten Intervall durchgeführt werden. Typ-II-Endoleaks mit assoziiertem Wachstum des Aneurysmasacks können durch Embolisation der verantwortlichen Gefäße behandelt werden. Ob eine Behandlung immer durchgeführt werden *muss,* ist umstritten. Eine Behandlungsindikation von einem Typ-II-Endoleak mit wachsendem Aneurysmasack ist jedoch gegeben, wenn durch eine Verkürzung des Aneurysmahalses ein sekundäres Typ-I-Endoleak droht. Typ-I-Endoleaks stellen die Hauptlimitation der Stentgraft-Therapie dar. Die beste Prävention eines Typ-I-Endoleaks ist die Bereitstellung einer adäquaten proximalen Landezone. Dies kann durchaus bedeuten, dass fenestrierte Stentgrafts verwendet werden müssen. Die Verwendung von Schrauben oder anderen Fixationsinstrumenten zur sicheren Behandlung auch kurzer Hälse ist derzeit noch in der Studienphase.

## Abstract (en)

All patients who underwent endovascular aortic repair (EVAR) need a thorough follow-up, at least yearly. Contrast-enhanced ultrasound and computed tomography angiography (CTA) are the most important modalities for detection of endoleaks, whereby CTA allows better differentiation of endoleak type. High pressure endoleaks (type I and III) are an absolute indication for treatment if they do not resolve spontaneously in the short term. Type II endoleaks are mostly benign and may be routinely controlled if there is no progression of the aneurysm. Type II endoleaks associated with aneurysm progression may be treated with embolization; however, whether they *must* be treated is a matter of discussion. Nonetheless, a type II endoleak must be treated when progression shortens the aneurysm neck and the threat of a type I endoleak is at hand. Type I endoleaks are the main limitation of stent grafts. An adequate proximal landing zone is the best prevention for type I endoleaks, even if fenestrated stent grafts have to be used. Various fixation devices for short necks are currently under investigation.

Seit der Erstbeschreibung der endovaskulären Aortenreparatur (EVAR) des abdominellen Aortenaneurysmas hat diese Methode eine signifikante Verschiebung der Patientenströme und Änderung der Behandlungskonzepte bewirkt [46, 54]. Eine Vielfalt von Endoprothesen ist gekommen und gegangen und, die Prothesen der jüngsten Generation weisen über alle Hersteller hinweg hohe technische Erfolgsraten, große Patientensicherheit und gute Langzeitstabilität auf [14, 25, 26, 41]. Im Vergleich zur offenen Operation ist die endovaskuläre Methode wesentlich weniger invasiv mit verkürztem Krankenhausaufenthalt, früherer Mobilisierung, geringeren perioperativen Beeinträchtigungen und besserer Kurzzeitmortalität [8, 29, 40]. Diese Verbesserungen kommen auf Kosten der Notwendigkeit einer, im Vergleich zur offenen Operation, engmaschigeren Nachsorge und höheren Rate von Undichtigkeiten, den sog. Endoleaks [1, 2]. Diese Übersichtsarbeit behandelt die Möglichkeiten der Prävention, Diagnose, Differenzialdiagnose sowie der endovaskulären Therapie dieser Endoleaks und zeigt die Ergebnisse dieser sekundären Korrekturverfahren.

Als Endoleak nach endovaskulärer Behandlung eines Aortenaneurysmas wird der Übertritt von Blut in den Aneurysmasack bezeichnet, der nach dem Setzen eines Stentgrafts theoretisch vollständig von der Perfusion exkludiert sein sollte [10]. Je nach Lokalisation des Ursprungs des weiterbestehenden Zuflusses werden fünf verschiedene Typen von Endoleaks mit assoziierten Subgruppen unterschieden (Abb. 1; [35]).

**Figure 1:**
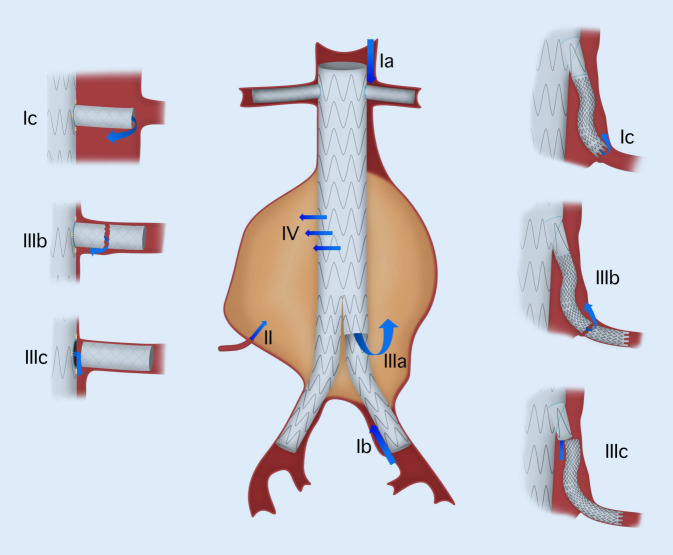

## Typ I.

Beim Typ-I-Endoleak liegt eine Restperfusion außerhalb des Stentgrafts an seiner proximalen (Ia) oder distalen (Ib) Abdichtungszone, oder an einem Seitenast bei fenestrierten oder verzweigten Stentgrafts (Ic) vor [35].

## Typ II.

Das Typ-II-Endoleak definiert sich durch eine Perfusion des Aneurysmasacks durch retrograden Fluss aus den Lumbalarterien oder der A. mesenterica inferior (AMI; [46]). Zudem können verschiedene Gefäße auch Einstrom und Ausstrom aus dem Aneurysmasack ermöglichen [1].

## Typ III.

Das Typ-III-Endoleak entsteht aus Undichtigkeiten zwischen einzelnen Komponenten der Endoprothese, oder der Prothese selbst: Man unterscheidet zwischen aortoaortalen bzw. aortoiliakalen Komponentenundichtigkeiten (IIIa), z. B. durch Dislokation (Herausgleiten einer Komponente aus dem Hauptkörper), Membraneinrissen oder Stentfrakturen (IIIb), sowie Undichtigkeiten zwischen den seitlichen Verzweigungen und den Verbindungsstentgrafts (IIIc) [35, 42].

## Typ IV.

Das Typ-IV-Endoleak ist definiert als eine Perfusion des Aneurysmasacks in Folge einer Porosität des Prothesenmaterials [51]. Insbesondere die dünnwandigen Materialien moderner Niedrigprofilprothesen können (initial) eine Perfusion im Sinne eines Typ-IV-Endoleaks zeigen.

## Typ V.

Das Typ-V-Endoleak wird auch als Endotension bezeichnet und ist definiert als eine fortgesetzte, durch Zunahme des Durchmessers des Aneurysmasacks ohne Nachweis eines der anderen Endoleaks Typ I–IV [27]. Manche Arbeitsgruppen beschreiben ultrafiltriertes Plasma (im Gegensatz zu Blut mit zellulären Anteilen) als Inhalt des Aneurysmasacks [57]. Als Ursache kommen mechanische (Pulsationen), osmotische oder andere noch nicht gänzlich erforschte Mechanismen in Frage [47, 48].

Die Endoleaks sind die häufigste Komplikation nach EVAR und stellen einen wesentlichen Risikofaktor für eine postinterventionelle Ruptur des Aneurysmasacks dar [32]. Aus diesem Grund sind engmaschige bildgebende Verlaufskontrollen der Patienten nach EVAR ein wichtiger Bestandteil der Nachsorge. So sehen sowohl die aktuellen amerikanischen wie auch die europäischen Guidelines eine erste bildgebende Kontrolle mittels Computertomographie-Angiographie (CTA) 1 Monat nach EVAR vor [11, 55]. Nach beiden Guidelines soll ein Typ-I- oder ein Typ-III-Endoleak unmittelbar einer Behandlung zugeführt werden, während ein Typ-II-Endoleak im Intervall kontrolliert werden soll. Hier hören jedoch die Gemeinsamkeiten bereits auf, denn die europäischen Guidelines empfehlen eine Kontrolle nach 12 Monaten, die amerikanischen Guidelines bereits nach 6 Monaten, obwohl Go et al. schon 2008 gezeigt haben, dass eine weitere Verlaufskontrolle nach einem Jahr ausreichend ist. Die europäischen Guidelines differenzieren die Typ-II-Endoleaks bei der 1‑Jahres-Kontrolle je nach Änderung des Aneurysmadurchmessers weiter und sehen dann entweder eine Reintervention bei Wachstum des Aneurysmasacks um mehr als 1 cm, eine weitere jährliche Kontrolle bei größenkonstantem Aneurysmasack oder eine CTA-Kontrolle in 5 Jahren bei Schrumpfung des Aneurysmasacks um mehr als 1 cm vor. Bei Abwesenheit eines Endoleaks in der initialen Nachsorge Untersuchung nach einem Monat sollen die Kontrollen nach den amerikanischen Guidelines jährlich erfolgen, nach den europäischen Guidelines nur alle 5 Jahre. Letzteres erscheint doch recht lange, vor allem, wenn man bedenkt, dass insbesondere jene Endoleaks, die unmittelbar postinterventionell nicht vorhanden waren, aber frühestens ein Jahr nach EVAR auftreten, meistens eine Reintervention erfordern und mit einem großen Durchmesser assoziiert sind [39].

Aber auch bzgl. der bildgebenden Modalität unterscheiden sich die Leitlinien: So wird bei den amerikanischen Guidelines der Ultraschall (US) mit Ausnahme der ersten Nachuntersuchung gleichwertig zur CTA gewertet, während die europäischen Leitlinien sogar den US vorrangig empfehlen, vor allem für die jährlichen Verlaufskontrollen, während die CTA nur bei den Kontrollen alle 5 Jahre zum Einsatz kommen soll. In diesem Bereich kann insbesondere der kontrastmittelverstärkte Ultraschall punkten, der bei deutlich niedrigeren Kosten eine höhere Sensitivität zur Detektion von Endoleaks als die CTA aufweist, insbesondere bei Typ-II-Endoleaks [18, 22, 50, 56].

Im Bereich der CTA haben sich durch die Dual-Energy-Technik neue Möglichkeiten ergeben; so kann entweder die notwendige Strahlenexposition erheblich reduziert werden oder durch monoenergetische Rekonstruktionen die Sensitivität für die Detektion von Endoleaks deutlich verbessert werden [34, 43].

**Figure 2:**
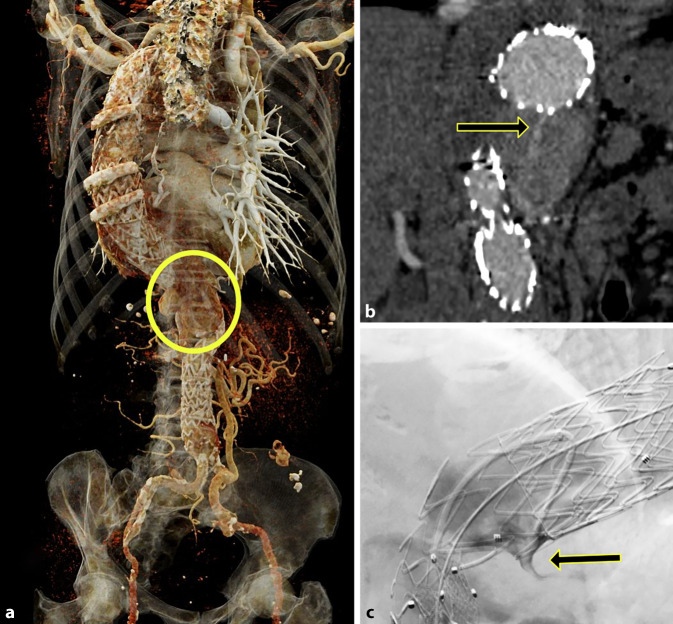

Der größte Vorteil der CTA besteht jedoch in der besseren Übersicht zur Planung einer Reintervention, sodass in einigen Zentren weiterhin die CTA die präferierte Nachsorge-Untersuchung darstellt. Gerade bei komplexen Stentgraft-Designs (z. B. vierfach gebranchter Stentgraft am throakoabdominellen Übergang mit Seitenästen für die Viszeral-und Nierenarterien) kann es mitunter schwierig sein, nicht nur den Endoleak-Typ, sondern auch die Quelle des Endoleaks präzise zu bestimmen. Durch die Verwendung von dynamischen CT-Akquisitionen kann der Blutfluss mit einer sehr hohen zeitlichen Auflösung dargestellt werden, bei Begrenzung des Scan-Rangs auf die Detektorbreite ist das untere Limit durch die Rotationszeit der Röhre gegeben und liegt im Bereich von 250 bis 300 ms. Die Möglichkeiten dieser Technik wurden in einem Fallbericht bereits 2010 erstmals unter Beweis gestellt [5]. Die erste Kohortenstudie dazu folgte 2014, diese zeigte jedoch, dass bei 8 Endoleaks (1 Typ I, 6 Typ II und 1 Typ III) in 39 Patienten eine routinemäßige Anwendung der Technik aufgrund der hohen Strahlenexposition nicht sinnvoll ist [36]. Aus diesem Grund läuft seit 2016 an der Medizinischen Universität Wien eine prospektive Studie, bei der nur Patienten mit einem unklaren Endoleak nach einer Standard-CTA eingeschlossen werden. Bis dato konnten über 20 Patienten eingeschlossen werden, und in nahezu allen Fällen konnte anhand der dynamischen CTA die anschließende Intervention mit besserer Planung erfolgen (Abb. 2). Dies bringt mehrere Vorteile mit sich: So müssen angiographisch nicht alle Äste einer komplexen Prothese sondiert und kontrolliert werden, damit besteht ein geringeres Risiko durch Manipulation die Prothese bzw. die Verbindungsstellen der einzelnen Komponenten zu beschädigen, außerdem wird weniger Kontrastmittel benötigt. Die Dosiseinsparung an ionisierender Strahlung während der Angiographie kommt nicht nur dem Patienten, sondern auch dem interventionellen Team im Eingriffsraum zugute.

## Typ-I-Endoleak

### Diagnose

Intraprozedural sind Ia-Endoleaks in der Abschlussangiographie als frühe Kontrastmittel(KM)-Anreicherung im Aneurysmasack erkennbar, oftmals pulsatil und pulssynchron. Die Abgrenzung zum Typ-III- oder zum Typ-IV-Endoleak kann durch Katheterinjektion oberhalb und innerhalb des Stentgrafts (unter Verhinderung eines Refluxes von Kontrastmittel in die Aorta oberhalb des Stentgrafts) gemacht werden: Ein Typ-IV-Endoleak würde beispielsweise sowohl bei Injektion oberhalb als auch innerhalb des Stentgrafts eine Anfärbung verursachen, das Typ-Ia-Endoleak nicht. Diese Form der Diagnose hat jedoch (speziell bei mehreren Korrekturen im Rahmen der Primärimplantation) ihre Grenzen aufgrund der Kontrastmittel und Strahlungsdosis.

Späte Typ-I-Endoleaks (im Rahmen der Verlaufskontrollen) werden zumeist in der CT diagnostiziert. Differenzialdiagnostisch geben hier nicht nur die Kontrastmittel, sondern auch andere morphologische Kriterien Hinweise: Expansion des Halses über den nominellen Durchmesser des Stentgrafts, Verkürzung des Halses durch Vergrößerung des Aneurysmasacks nach kranial oder die Detektion eines Kontrastmittelsaums zwischen dem äußeren Rand des Stentgrafts und dem inneren Rand der Aorta auf Höhe der proximalen Abdichtungszone sind differenzialdiagnostische Kriterien. Wenn die Abgrenzung zwischen einem Typ-I- und einem Typ-II-Endoleak nicht gelingt, dann muss entweder eine diagnostische Angiographie oder aber eine neuentwickelte zeitlich hochaufgelöste computertomographische Spezialuntersuchung durchgeführt werden.

### Behandlung

**Figure 3:**
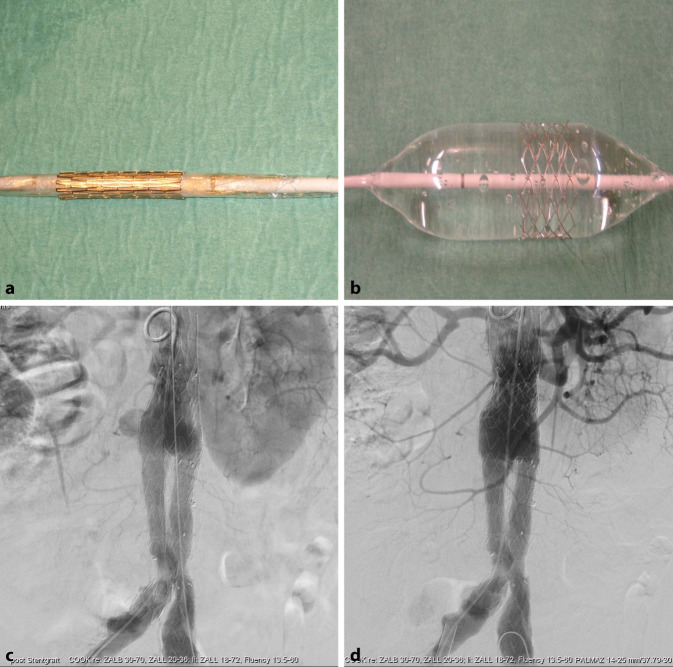

Intraprozedural wird ein Ia-Endoleak primär mittels Ballonangioplastie therapiert. Ist diese Methode erfolglos, kann die Radialkraft dauerhaft durch die Implantation eines ballonexpandierbaren Metallstents (Palmaz, Cordis) erhöht werden. Dieser muss vom Operateur selbst auf einen geeigneten Ballon (Ösophagusdilatations- oder Valvuloplastieballon) montiert werden und erfordert eine besondere Technik, um Dislokationen zu vermeiden (Abb. 3). Bei Hälsen mit großem Durchmesser wird dieser Stent außerhalb der empfohlenen Durchmesser verwendet und muss eventuell zusätzlich mit einem Latexballon nachdilatiert werden. Oft ist nicht vermeidbar, dass einige Stentdrähte über die Ostien der Nierenarterien reichen. Der Operateur muss sich bewusst sein, dass er hier zusätzliche Hindernisse für eine mögliche Verlängerung mit einer fenestrierten Endoprothese schafft.

Reicht der primär implantierte Stentgraft nicht ganz an die Nierenarterien heran, kann mit einem kurzen tubulären Segment (einem sog. Cuff) das Endoleak versorgt werden. Je nach räumlichen Verhältnissen können hier selbstexpandierende Verlängerungen oder kurze großlumige Ballon-expandierbare Stentgrafts (z. B. BeGraft Aortic, Bentley, Hechingen) verwendet werden. Weitere Therapiemethoden zur Abdichtung eines Ia-Endoleaks ohne proximale Verlängerung sind endovaskulär applizierbare Schrauben (Aptus Endosystems, Medtronic, Minneapolis, USA; [28]). Mit diesem System können von einem femoralen Zugang durch eine spezielle steuerbare Schleuse bis zu 10 endovaskulär applizierbare, etwa 3 × 5 mm große Schrauben radiär durch den proximalen Rand des Stentgrafts in die Aortenwand gesetzt werden und potenziell die Apposition verbessern. Einige Autoren empfehlen schließlich die kathetergestützte Embolisation des Spalts zwischen Stent und Aorta mit verschiedenen Materialien. Die beiden letztgenannten Methoden sind in der Erprobungsphase und haben in der Institution der Autoren keine durchgehenden Erfolge gezeigt.

Die nach der Meinung der Autoren beste Methode zur Behandlung eines Typ-Ia-Endoleaks stellt eine signifikante Verlängerung des Stentgrafts nach proximal unter Verwendung einer 2‑ bis 4fach fenestrierten tubulären Prothese dar [15]. Hier kann speziell bei initial grenzwertig kurzen Hälsen eine sichere Landung in einem *gesunden* Aortensegment erfolgen und – im Fall der vierfach fenestrierten Prothese – steht auch eine Landezone für eine allfällig später notwendige noch weitere proximale Verlängerung mit weiteren Stentgrafts zur Verfügung. Um eine sichere Landung des tubulären Segments in der primär implantierten Endoprothese zu ermöglichen, wäre es von Vorteil, im Fall der Implantation einer infrarenalen Prothese bei grenzwertig kurzen Hälsen bereits primär ein Fabrikat mit einem längeren Hauptkörper zu verwenden (Cook Medical, Bloomington, USA, Endologix, Irvine, USA), da die tubuläre Verlängerung eines üblicherweise 3–4 cm langen Hauptkörpers wiederum das Risiko eines sekundären Typ-III-Endoleaks in sich birgt. Nachteile der fenestrierten Verlängerung sind zweifelslos die lange Lieferzeit und die hohen Kosten. Beim elektiven Patienten muss vor einer aufwendigen Therapie eines Typ-Ia-Endoleaks bedacht werden, dass in einzelnen kleinen Serien im Rahmen kurz- bis mittelfristiger Beobachtungen Typ-Ia-Endoleaks auch eine relativ hohe spontane Verschlussrate aufwiesen und diese aufwendige Therapie folglich nur für persistierende Ia-Endoleaks zur Anwendung kommen sollte [53].

Die Behandlung von Typ-Ib-Endoleaks stellt sich üblicherweise einfacher dar als von Typ-Ia-Endoleaks. Eine Verlängerung des distalen Stentschenkels in die A. iliaca externa kann durch Embolisation einer A. iliaca interna oder durch Implantation einer iliakalen Bifurkationsprothese erfolgen. Da auch die unilaterale Embolisation einer A. iliaca interna bei aktiven Menschen in bis zu 30 % zu einer permanenten glutealen Claudicatio führt, wird an der Institution der Autoren bei jüngeren und aktiveren Menschen die Iliac-side-branch-Implantation bevorzugt [19]. Die sekundäre Implantation eines Iliac side branch nach endovaskulärer Aortenversorgung ist aufgrund der fehlenden Möglichkeit eines kontralateralen Zugangs etwas aufwendiger und erfordert zumeist einen brachialen Zugang für die Implantation des A.-iliaca-externa-Beinchens. Eine rein unilateral-femorale Implantation ist durch die Verwendung moderner steuerbarer Schleusen in Einzelfällen schon gelungen [30].

### Prävention

Nach Ansicht der Autoren ist die wichtigste Maßnahme der Prävention die Bereitstellung einer adäquaten proximalen Landezone. Seit der weiträumigen Verbreitung der endovaskulären Aortentherapie wurden immer wieder die von den Herstellern und von den gängigen Studien vorgegebenen Limitationen im Hinblick auf Länge, Durchmesser, Angulation, Verkalkung und Thrombosierung des proximalen Aneurysmahalses umgangen, im Versuch, die Methode auch grenzwertigen, sog. *hostilen* Hälsen zugänglich zu machen. Wiewohl dies zur Weiterentwicklung der Technik beitragen kann, so sollte angesichts der mittlerweile in Westeuropa weiträumig verfügbaren Methode des fEVAR (fenestrierte EVAR) dieser bei grenzwertigen Hälsen der Vorrang gegeben werden. Analog dazu stellt sich die Frage, ob speziell bei jungen und aktiven Patienten eine distale Landung mit einem 24 mm großen iliakalen Bein in der oberhalb der A. iliaca interna sinnvoll ist oder hier nicht gleich einer iliakalen Bifurkationsprothese der Vorrang gegeben wird.

## Typ-II-Endoleak

### Diagnose

Das Typ-II-Endoleak stellt die häufigste Form des Endoleaks nach EVAR dar [38]. Intraprozedural kann ein lumbales Typ-II-Endoleak klassisch als nach Kontrastmittelinjektion verzögerte retrograde Füllung des Aneurysmasacks über die Lumbalarterien oder die AMI nach Füllung der jeweiligen zuführenden Gefäße (A. iliaca interna – lumbales Kollateralnetzwerk oder A. mesenterica superior – Riolan-Anastomose) gesehen oder diagnostiziert werden. In der CT zeigt sich oftmals eine in der venösen Phase verstärkte Füllung des Aneurysmasacks mit Punctum maximum über einer oder mehreren Lumbalarterien oder der Einmündung der AMI. Wenn diese Füllungen keinen direkten Kontakt zur Außenwand des Stentgrafts haben, ist die Differenzialdiagnose einfach; schwieriger ist es bei einer diffusen Füllung von größeren Arealen des Aneurysmasacks. Auch hier kann die zeitlich hoch aufgelöste CT durch Darstellung der Flussrichtung im Aneurysmasack differenzialdiagnostischen Aufschluss geben [6].

### Behandlung

Die Indikation zur Behandlung eines Typ-II-Endoleaks ergibt sich aus zweierlei Gründen: einerseits bei Wachstum des Aneurysmasacks aufgrund der Möglichkeit einer Ruptur direkt durch das Typ-II-Endoleak oder aber aufgrund der Gefahr der kontinuierlichen Verkürzung des Halses durch das Aneurysmawachstum und dem sekundären Auftreten eines Typ-Ia-Endoleaks, das dann seinerseits zur Ruptur oder Stentgraft-Dislokation führen kann [13, 49].

Typ-II-Endoleaks werden durch Embolisation der zuführenden Arterien behandelt, entweder transarteriell oder nach direkter Punktion des Aneurysmasacks [1].

**Figure 4:**
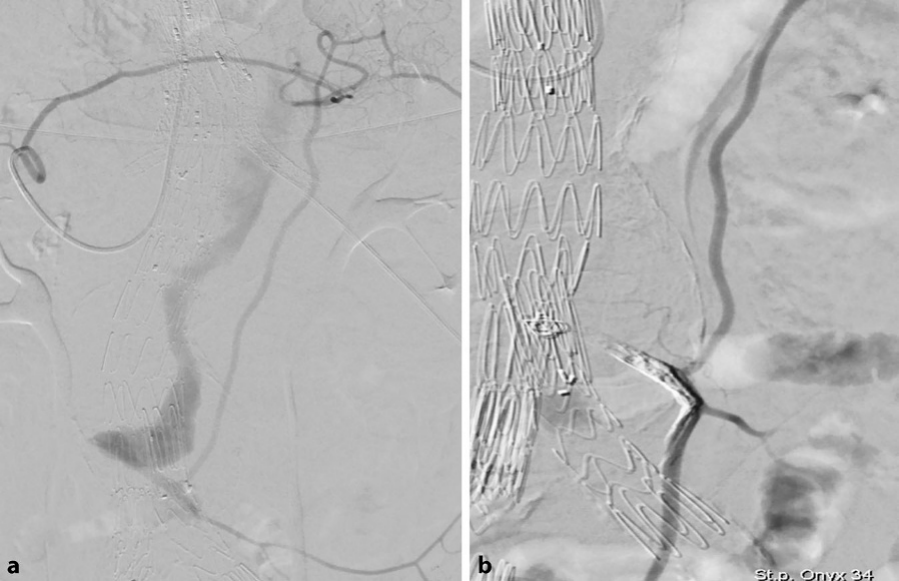

Die transarterielle Embolisation der dominanten speisenden arteriellen Gefäße über arterielle Anastomosen aus der A. mesenterica superior oder A. iliaca interna ist die häufigste Behandlungstechnik [1]. In den meisten Fällen kann diese Behandlung unter Verwendung eines Mikrokatheters in Lokalanästhesie perkutan von einem femoralen Zugang her durchgeführt werden. Für die Embolisation der AMI erfolgt der Zugang üblicherweise über die mesenteriumsnahe Riolan-Anastomose oder über die weiter peripher dem Kolon nahegelegene marginale Anastomose. Über eine Sicherung des Zugangs zur A. mesenterica superior üblicherweise mit einem Sidewinder-Katheter wird der Mikrokatheter nach angiographischer Verifikation in die meist proximal abgehende, anastomosespeisende Arkade der A. mesenterica superior eingelegt. Zu beachten ist insbesondere bei marginalen Anastomosen, dass ein ausreichend langer Mikrokatheter von 150–170 cm Länge verwendet werden sollte, um bei der Sondierung nicht feststellen zu müssen, dass die Katheterlänge nicht ausreicht. Wenn die Spitze des Mikrokatheters zwischen dem Abgang der AMI und ihrem ersten Ast liegt, kann eine Embolisation durchgeführt werden, wobei zu beachten ist (insbesondere bei Verwendung flüssiger Embolisate), einen Reflux in die abgehenden AMI-Äste unbedingt zu verhindern, um die Wirksamkeit der Anastomose nicht zu beinträchtigen und Ischämien im distalen Kolon zu verhindern (Abb. 4).

Analog dazu erfolgt die Embolisation über die A. iliaca interna, zumeist von einem kontralateralen Zugang unter Verwendung einer flexiblen längeren Schleuse. Nach Eingehen in die üblicherweise früh abgehende A. iliolumbalis, gilt es mit dem Mikrokatheter das Kollateralnetzwerk zu überwinden und die verantwortliche Lumbalarterie aufzusuchen, was vom Verlauf und Kaliber der kollateralen Äste abhängt und nicht immer gelingt. Üblicherweise ist die Sondierung umso schwerer, je höher die Lumbalarterie gelegen ist. Im Gegensatz zur AMI-Embolisation, bei der die Autoren Spiralen bevorzugen, ist die Verwendung von Flüssigembolisaten bei der Lumbalarterienembolisation unter Umständen hilfreich, um einen kontrollierten Reflux zum Stamm der Lumbalarterie durchzuführen, wenn der Katheter nicht ganz bis zum Ostium vorgeschoben werden kann. Hierbei ist peinlich genau in einer Subtraktionsangiographie auf die Möglichkeit der Kollateralen zur A. spinalis anterior zu achten! Auch die übermäßige Verwendung von Flüssigembolisat mit peripheren Embolisationen kann zu Hautnekrosen oder Muskelischämien führen.

Die Ergebnisse der Behandlung des Typ-II-Endoleaks mit transarterieller Embolisation zeigen klinische Erfolgsraten (kein weiteres Wachstum des Aneurysmasacks im Follow-up) zwischen 65 und 100 % [24, 33, 44, 58].

Alternativ kann bei fehlendem transarteriellem Zugang der Aneurysmasack direkt punktiert werden, wobei eine Kombination aus CT und Angiographie die ideale Modalität darstellt, aber nicht immer verfügbar ist. Eventuell kann anhand einer zuvor durchgeführten CT ein sicherer Punktionsweg identifiziert und in der Angiographie (oder mittels Kombination im CT) durchgeführt werden [1]. Nach Einstechen einer 20G-Koaxialnadel in den perfundierten Anteil des Aneurysmasacks kann oft ein pulsatiler Rückstrom beobachtet werden, worauf über einen Draht eine dünne Schleuse und ein kurzer Katheter in den Aneurysmasack eingelegt und sodann nach angiographischer Verifikation der Abgänge der verantwortlichen Arterie diese mit einem Mikrokatheter sondiert und embolisiert werden. Auch hier können mikrovaskuläre Plaques, Spiralen oder flüssige Embolisate verwendet werden. Wenn die Sondierung des zuführenden Gefäßes nicht gelingt, oder zusätzlich zu einer selektiven Embolisation des zuführenden Gefäßes, kann beim Rückzug des Katheters auch der perfundierte Anteil des Aneurysmasackes direkt embolisiert werden. Einige Serien haben hohe technische und klinische Erfolgsraten nach derartigen transabdominellen Embolisationen beschrieben [9, 59].

Mehrere rezente Metaanalysen haben das Paradigma der Behandlungsindikation des Typ-II-Endoleaks bei wachsendem Aneurysmasack in Frage gestellt: Antoniou et al. beschreiben in einer Metaanalyse 16.974 EVAR-Eingriffe mit 152 Rupturen, nach einer durchschnittlichen Zeit zwischen 16 und 50 Monaten. Von diesen waren 14 Rupturen auf ein Typ-II-Endoleak zurückzuführen [3].

Charisis et al. beschreiben in einer Metaanalyse 2643 Typ-II-Endoleaks [12], wobei nicht alle Parameter in jeder Studie untersucht wurden. Von den 1496 Typ-II-Endoleaks sistierten 730 (48,8 %); 343 von 852 (40,2 %) persistierten und 313 von 1075 (29 %) führten zu einem Wachstum des Aneurysmasacks. Die Rupturrate betrug im Mittel 1,1 % der Typ-II-Endoleaks (Spannweite: 0–5,3 %). Wenn auch derzeit keine explizite Analyse der Rupturrate des unbehandelten Typ-II-Endoleaks bei wachsendem Aneurysmasack existiert, so kann aus den vorgenannten Studien doch geschlossen werden, dass selbst bei wachsendem Sack die Ruptur sehr selten ist. Hinsichtlich der Indikationsstellung zu einer interventionellen Behandlung ergibt sich daraus, dass jegliche Behandlung eine sehr hohe Erfolgsrate und eine sehr niedrige Komplikationsrate aufweisen muss, um ethisch gerechtfertigt zu sein. Alternativ könnte auch argumentiert werden, Typ-II-Endoleaks selbst bei wachsendem Aneurysmasack zu kontrollieren, um eine Progression zu einem Typ-I-Endoleak durch Verkürzung des proximalen oder distalen Halses auszuschließen [12].

### Prävention

Eine Möglichkeit der Prävention von Typ-II-Endoleaks stellt die präoperative Embolisation kaliberstarker Lumbalarterien oder der AMI dar. Über die Effektivität existieren nur beschränkte Daten. Biancari et al. zeigten, dass die Endoleak-Rate nach präoperativer Embolisation der AMI etwa halbiert wurde, allerdings ist aufgrund der relativ geringen Prävalenz des Typ-II-Endoleaks von 20 % eine hohe „number to treat“ bei der präoperativen Embolisation erforderlich [7]. Dies spricht auch nach Meinung dieses Autors gegen eine grundsätzliche präoperative AMI-Embolisation. In der Institution des Autors werden Typ-II-Endoleaks mit wachsendem Aneurysmasack grundsätzlich embolisiert und bei fehlendem Erfolg kontrolliert. Die Ultima Ratio der Behandlung des Typ-II-Endoleaks ist die offen-chirurgische oder laparoskopische Präparation des Aneurysmasacks und Ligatur oder Clippung der zuführenden Arterien [52]. Diese wird aufgrund der Invasivität nur bei drohenden Komplikationen durchgeführt.

## Typ-III-Endoleak

Das Typ-III-Endoleak ist definiert als Komponentenseparation eines modularen Stentgraft-Systems (IIIa) oder ein Defekt in der Membran (IIIb) nach Chaikof, zudem wird ein Typ IIIc unterschieden, wenn die Komponentenseparation einen Verbindungsstentgraft bzw. die Verzweigung am Hauptkörper betrifft [10]. In den letzten Jahren wurde angesichts der wachsenden Prävalenz von fenestrierten und verzweigten Prothesen der Typ IIIc als Diskonnektion zwischen Fenestrierung/Verzweigung und Verbindungsstentgraft definiert [35]. Die Autoren haben beobachtet, dass Defekte in Verbindungsstentgrafts, insbesondere Membrandefekte im Sinne eines Typ-IIIb-Endoleaks typischerweise mit gängigen CT-Protokollen schwer detektierbar sind, in einzelnen Patienten gehäuft auftreten und bei stark angulierten Verbindungsstentgrafts wahrscheinlicher sind [31].

### Diagnose

In der CT zeigt sich das Typ-III-Endoleak typischerweise als eine rasche Anfärbung im Aneurysmasack mit breitem Kontakt zum Stentgraft, dennoch kann eine Abgrenzung zu den anderen Endoleak-Typen schwierig sein. Aufgrund der vom Untersucher angenommenen Seltenheit von Membrandefekten werden solche üblicherweise erst nach einer selektiven Angiographie mit Kontrastmitteljet oder Drahtpassage durch die Membran nachgewiesen [23, 31]. Membrandefekte treten sowohl nach Manipulationen (insbesondere Implantation von ballonexpandierbaren Stents im Stentgraft), aber auch spontan auf (Abb. 2 und 5).

**Figure 5:**
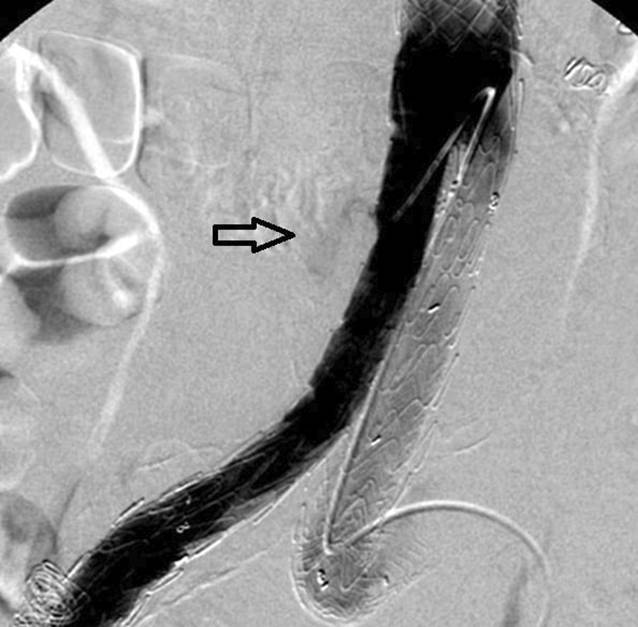

### Behandlung

Ein Typ-III-Endoleak wird durch die erneute Implantation eines Stentgrafts über die defekte Stelle behandelt. Je nach Lokalisation des Defekts kann dies aber auch schwierig oder unmöglich sein. Ein Endoleak unmittelbar an der Bifurkation einer abdominellen Endoprothese kann die Implantation eines aortomonoiliakalen Systems mit Verschluss der kontralateralen Seite und Versorgung eines Crossover-Bypass notwendig machen oder die Implantation einer speziellen Bifurkationsendoprothese mit invertiertem kontralateralem Bein, um in der begrenzten Höhe zwischen Nierenarterien und Neobifurkation die Konnektion mit der Gegenseite zu ermöglichen (Cook Medical, Bloomington, Indiana).

### Prävention

Die Diskonnektion zwischen der Bifurkationsprothese und dem iliakalen Bein wird von einzelnen Herstellern durch ein spezielles Widerhackensystem verhindert (Treo, Terumo Aortic, Japan). Um eine Spannung zwischen iliakalem Schenkel und Hauptkörper bei stark gekrümmten und elongierten Gefäßen zu vermeiden, ist auf eine ausreichende Länge des entsprechenden iliakalen Schenkels zu achten, der sich auch nach Rückzug des steifen Führungsdrahts ohne übermäßige Verkürzung dem Gefäßverlauf anpassen kann.

Bei fenestrierten und verzweigten Endoprothesen waren Verbindungsstentgrafts mit einschichtiger Membran häufiger die Quelle von Typ-III-Endoleaks [45].

## Typ-IV-Endoleaks

Durch die Entwicklung und kontinuierliche Verbesserung des Graftmaterials wurden Typ-IV-Endoleaks in den moderneren Stentgraft-Systemen selten beobachtet [46]. Die Detektionsrate des Typ-IV-Endoleaks hängt auch von der Qualität der Angiographie-Einheit ab [16, 17, 20]. Mit einem C‑Bogen und Handinjektion werden solche Endoleaks kaum zu erkennen sein. Persistierende Typ-IV-Endoleaks werden kaum beobachtet. Mit dem rezenten Trend zu immer kleinkalibrigeren Applikationsbestecken ging in einigen Fabrikaten allerdings auch eine Durchmesserreduktion sowohl der Stents als auch der Gewebedicke einher. Nach Erfahrung der Autoren zeigen speziell Stentgrafts mit ultradünnen Applikatoren im Bereich von 14–16 F temporäre Typ-IV-Endoleaks, speziell bei komplexen Prozeduren mit hohen erforderlichen Heparindosen sind derartige Endoleaks zu beobachten.

## Prävention und Therapie

Nachdem Typ-IV-Endoleaks in der überwältigenden Mehrzahl der Fälle selbstlimitierend sind, stellt sich üblicherweise keine spezifische Behandlungsindikation [21, 51]. Allerdings kann im Fall einer akuten Aortenruptur der persistierende Volumenverlust durch ein Typ-IV-Endoleak die hämodynamische Stabilität des Patienten signifikant beinträchtigen. Nach der Meinung der Autoren ist es in diesem Fall sinnvoll, primär keine Systeme mit ultradünnen Applikatoren zu verwenden und im Falle des Falles (bei persistierender, lebensbedrohlicher hämodynamischer Instabilität) zum sicheren Ausschluss von anderen Typen von Endoleaks einen zweiten Stentgraft in den zuerst gesetzten zu implantieren [4]. In akut rupturierten Patienten kann nach massiver Transfusionsgabe ein Fehlen von Gerinnungsfaktoren die Ausbildung eines Typ-IV-Endoleaks potenziell begünstigen.

## Typ-V-Endoleaks

Typ-V-Endoleaks entstehen durch den undefinierten Begriff der *Endotension*. Bei diesem Endoleak kann mit der gegenwärtigen Bildgebung keine direkte Kontrastmittelextravasation nachgewiesen werden, obwohl ein Wachstum des Aneurysmasacks besteht. Als Ursache wurden die unterschiedlichsten Mechanismen vorgeschlagen, von extrem langsamem Blutfluss im Sinne eines nicht detektierten Endoleaks anderer Genese über Ultrafiltration des Blutes und Extravasation von Plasma bis hin zu subklinischer Infektion [27]. Bislang wurde kein erhöhtes Rupturrisiko beim Typ-V-Endoleak beschrieben [37]. Eine Behandlung wird daher erst bei sekundären Symptomen durch die raumfordernde Wirkung des wachsenden Aneurysmasacks zu indizieren sein.

## Fazit für die Praxis

- Endoleaks stellen eine wesentliche Limitation der endovaskulären Aortentherapie dar und machen die dauerhafte Nachsorge nach Stentgraft-Implantation notwendig.
- Die Computertomographie-Angiographie (CTA) und der kontrastmittelverstärkte Ultraschall stellen geeignete Modalitäten dar.
- Während Hochdruck-Endoleaks einer Therapie bedürfen, die in der Mehrzahl der Fälle mit endovaskulären Methoden möglich ist, können Typ-II-Endoleaks ohne Wachstum des Aneurysmasacks in Kontrolle verbleiben.
- Die Behandlungsindikation beim Typ-II-Endoleak mit Wachstum des Aneurysmasacks wird derzeit diskutiert.
- Spätestens aber, wenn das Wachstum des Aneurysmasacks beim Typ-II-Endoleak die Gefahr einer Verkürzung des Halses oder einer Komponentendislokation bewirkt, müssen auch diese Endoleaks einer Behandlung zugeführt werden.
